# Committee on Veterinary Medicine at the Society for Medical Education: Skills Labs in Veterinary Medicine – a brief overview

**DOI:** 10.3205/zma001048

**Published:** 2016-08-15

**Authors:** Marc Dilly, Christian Gruber

**Affiliations:** 1 Stiftung Tierärztliche Hochschule Hannover, Clinical Skills Lab, Hannover, Germany; 2 Stiftung Tierärztliche Hochschule Hannover, BEST-VET, Hannover, Germany

**Keywords:** Skills Lab, Simulators, Simulation-based Training, Clinical Skills Training, Veterinary Medical Education

## Abstract

Since 2012, skills labs have been set up to teach practical skills at veterinary training facilities in the German-speaking world. In addition to didactic considerations, ethical points of view in terms of animal protection form the basis of the increasing significance of skills labs in veterinary medicine. Not least because of the quality standards in veterinary medicine training which apply across Europe, the link between veterinary medicine training facilities is particularly significant when it comes to the setting up and development of skills labs. The Committee on Veterinary Medicine is therefore not only interested in exchange and cooperation within veterinary medicine, but also sees an opportunity for mutual gain in the link with the Society for Medical Education Committee “Practical Skills”.

## Zusammenfassung

An den tiermedizinischen Ausbildungsstätten im deutschsprachigen Raum wurden seit 2012 Skills Labs für die Vermittlung praktischer Fertigkeiten eingerichtet. Neben didaktischen Überlegungen sind auch ethische Gesichtspunkte im Hinblick auf den Tierschutz die Grundlage für die zunehmende Bedeutung von Skills Labs in der Veterinärmedizin. Nicht zuletzt durch europaweit geltende Qualitätsnormen der tiermedizinischen Ausbildung ist die Vernetzung zwischen den tiermedizinischen Ausbildungsstätten von großer Bedeutung für die Einrichtung und Weiterentwicklung von Skills Labs. Der Ausschuss Tiermedizin ist daher nicht nur an dem Austausch und Kooperationen innerhalb der Veterinärmedizin interessiert, sondern sieht auch in der Vernetzung mit dem GMA-Ausschuss „Praktische Fertigkeiten“ die Möglichkeit der gegenseitigen Bereicherung.

## Introducing and presenting the Society for Medical Education Committee on Veterinary Medicine

The Society for Medical Education Committee on Veterinary Medicine includes (“officially” as it were) all members of the Society for Medical Education from veterinary medicine and is therefore an exceptional case (along with the Committee on Dentistry) compared to the other committees, the majority of which focus on specific content, as people are assigned to the committee based on their profession. In this sense, the committee takes on all topics that are of national significance for the seven veterinary medicine training facilities (Berlin, Gießen, Hanover, Leipzig, Munich, Vetsuisse and Vienna) in the German-speaking world.

Accordingly, there are links in terms of content to most committees and to the committee “Practical Skills”, because the students’ training in practical skills of course also occurs in veterinary medicine. Until recently, the intensive practical training took place almost exclusively on the animal or on (parts of) the cadaver. This type of training is not only extremely resource-intensive but is also being increasingly questioned from a didactic perspective and from both an ethical and animal protection-related perspective. As a result, in 2012 what are known as “skills labs” were created at various locations in the German-speaking world [3], [4] inspired by the international models which primarily come from the English-speaking world in which skills labs have been part of the day-to-day in (veterinary) medical training for some time. There are currently centrally established skills labs at five veterinary medical training facilities in Germany, Austria and Switzerland: Vienna, Hanover, Leipzig, Gießen and Bern. 

## Skills labs in veterinary medical training – the same and yet different

In human medicine in Germany and other countries there are now national catalogues of learning objectives while teaching at veterinary medical training facilities in Europe is organised at a supranational level and is subject to a uniform quality standard set down by the European Commission and is checked at regular intervals by visits carried out on behalf of the European Commission. The core content of the Europe-wide uniform quality standards are what are known as the “day one skills”, in other words skills and abilities that the students must have obtained by the end of their training and therefore by the “first day of their professional lives” [http://www.eaeve.org/fileadmin/downloads/sop/SOP_Annex4to8_Hanover09.pdf checked on 30 October 2015]. From a conceptual perspective they therefore correspond to what are known as the Entrusted Professional Activities (EPAs) in human medical training [2]. 

At around 50% of all of the skills listed in the day-one skills, practical skills make up the largest group by far and are therefore the focus of the training, leading to an increasing significance of the skills labs. 

Like in human medicine, complex processes in everyday clinic work are a major challenge for students, requiring the use of simulators and models with varying degrees of complexity. This not only facilitates students’ ability to gain practical skills, but concerns and anxiety about making mistakes with serious consequences for the animal while gaining practical skills are able to be alleviated [8].

The low number of training facilities compared to those for human medicine and the even lower number of skills labs meant that the development and sourcing of models was often a hurdle when setting up skills labs, as models for various species of animal were needed and therefore it was understandably not possible to rely on human medicine models. The relatively small market in veterinary medicine meant that animal models of this kind, however, were difficult to obtain and/or expensive. The rise in the number of skills labs, however, means that an increasing number of models and simulators are available commercially or are being developed in-house [1], [5], [6], [7], [8], [9], [10], [11], [12].

The links between veterinary medical training facilities, such as the online group “Veterinary Clinical Skills & Simulation” in the network NOVICE (Network Of Veterinarians In Continuing Education; http://www.noviceproject.eu/), founded in 2010 and dedicated exclusively to the teaching of practical clinical skills, are particularly helpful. The group is made up of more than 300 members from more than 30 countries.

In addition to links on online platforms, new meetings and conferences have also been established for exchange and the promotion of collaboration focusing on simulation-based teaching in veterinary medicine. The organisation “International Veterinary Simulation in Teaching” (InVeST, http://www.vetedsimulation.com), for example, was founded in 2011 and primarily looks at the development and validation of simulators and methods of communication in veterinary medical training. The first skills lab symposium in the Germany/Austria/Switzerland region took place in January 2014 in Hanover. 

## Collaboration of the committees

In both of the activities mentioned above – links via platforms and at thematically relevant conferences – the greatest opportunity for collaboration for the Veterinary Medicine Committee is with the Committee on Practical Skills.

Veterinary medicine has already been part of the simulator network established by the Committee on Practical Skills for two years. While there were hardly any contributions in the initial phase, participation has increased significantly in the past year and 29 models and simulators from veterinary medicine are now included in the network. More are to follow.

There is also an increasing presence of veterinary medicine at physical meetings in the form of conferences, so in the future the “International Skills Lab Symposium (iSLS)” organised by the Society for Medical Education Committee on Practical Skills will include contributions from veterinary medicine.

## Competing interests

The authors declare that they have no competing interests.
